# Structure–Function Relationship of the Most Abundant Ceramide Subspecies Studied on Monolayer Models Using GIXD and Langmuir Isotherms

**DOI:** 10.1021/acs.langmuir.5c01340

**Published:** 2025-05-27

**Authors:** Gerald Brezesinski, Lukáš Opálka, Chen Shen, Carolin Groetzsch, Emanuel Schneck, Adina Eichner

**Affiliations:** † 542351 Institute of Applied Dermatopharmacy at Martin Luther University Halle-Wittenberg, Weinbergweg 23, 06120 Halle (Saale), Germany; ‡ Institute for Condensed Matter Physics, Technical University of Darmstadt, Hochschulstr. 8, 64289 Darmstadt, Germany; § Skin Barrier Research Group, Faculty of Pharmacy, Charles University, Heyrovského 1203, 500 05 Hradec Králové, Czech Republic; ∥ 28332 Deutsches Elektronen-Synchrotron DESY, Notkestr. 85, 22607 Hamburg, Germany; ⊥ Department of Dermatology and Venereology, Martin Luther University Halle-Wittenberg, Ernst-Grube-Str. 40, 06120 Halle (Saale), Germany

## Abstract

The main lipid compounds of the outermost layer of human skin are ceramides (CERs), free fatty acids, and cholesterol. Although numerous studies performed in the past could demonstrate the importance of these lipids for an intact skin barrier function, knowledge about the impact of each single component on the lamellar lipid films is still lacking. Especially, the CERs are a very heterogeneous group with high relevance for a proper barrier. It was found that the reason for the high stability of the lamellae is related to the lipid structure and function, with the type and extent of interactions between the head groups of the individual CER subspecies being particularly important. Elucidating these at the molecular level could help us to understand CER phase behavior in general. Using grazing incidence X-ray diffraction and measurements of Langmuir isotherms, the current work investigated the lateral packing of the monolayers of different subclasses of C18:0 CERs at air–water interfaces, including phytosphingosine, sphingosine, and dihydrosphingosine CERs, all with either α-hydroxy and nonhydroxy *N*-acylated fatty acyl. We were able to observe clear effects of the minimal differences in the polar headgroup structures of the sphingoid bases, with respect to the number and position of hydroxyl groups and double bonds, on the CER arrangement regarding the compressibility and structure of the films they formed, revealing that the hydroxyl group at the C4 of the phytosphingosine CERs leads not only to the formation of a hydrogen bond network but also to a stable suprastructure, which might be of high benefit for the barrier properties of intact skin.

## Graphical abstract

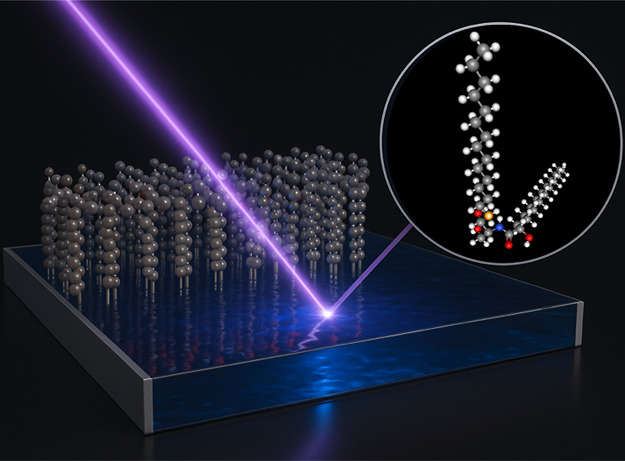

## Introduction

The outermost layer of human skin, the stratum corneum (SC), functions as the body’s most important barrier and consists of keratin-rich and avital corneocytes embedded in a complex lipid matrix. Changes in the composition of the lipid matrix are often associated with pathological changes in the skin. While in healthy skin, barrier damage is usually compensated by increased synthesis of the lipid components, this process is altered in skin diseases, which leads to an altered composition of the SC lipid matrix.^1–4^ The components of the lipid matrix are mainly ceramides (CERs), free fatty acids, and cholesterol,^5–7^ all with small head groups and a dominating hydrocarbon-based residue, resulting in strong lipophilic interactions.^8^ CERs are a very heterogeneous group of sphingolipids consisting of a sphingoid base *N*-acylated with a fatty acid. Up to now, 21 subclasses of free CERs were described to be found in human SC.^9^ They all differ in the number and position of the hydroxyl groups within their polar headgroup structure, which is the basis for the designation of the respective subclasses (see Figure 1).

**Figure 1:**
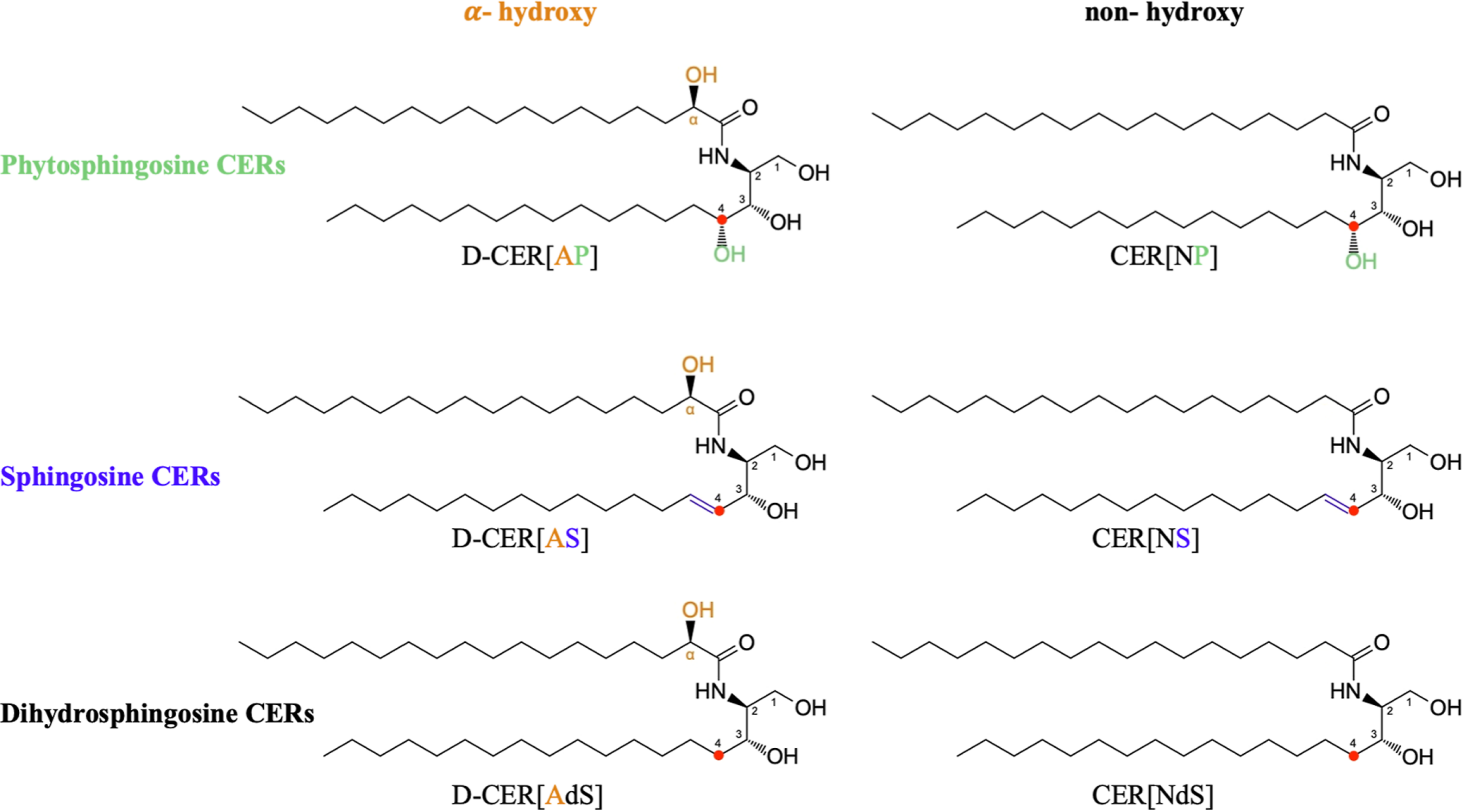
Chemical structures of the C18 CERs used. Following the nomenclature developed by Motta et al.,^10^ the naming of the CERs presented consists of two letters, the first describing the kind of the *N*-acylated fatty acid: “A” (α-hydroxy) with an OH group in the α-position or “N” (nonhydroxy), lacking an OH group in the α-position. The second letter describes the sphingoid bases with differences in the chemical structure at C4, marked with a red dot: sphingosine (S) with a double bond at C4, phytosphingosine (P) with an OH group at C4, or dihydrosphingosine (dS) with CH_2_ at C4. The d-conformation is connected to the chiral center at the fatty acyl chain (α-position label).

CER­[NP] and [AP] are two of the most abundant CERs in human SC, although their distribution differs in the literature, depending on the investigated skin localization and its conditions as well as the sensitivity of the analytical method chosen.^9,11–13^ So far, a specific function of each CER subclass to the SC barrier properties has not been assigned yet, although numerous sphingosine CERs and phytosphingosine CERs have been studied in the last decades applying SC lipid models to techniques like small- and wide-angle X-ray scattering,^14,15^ neutron diffraction,^16–21^ deuterium nuclear magnetic resonance (^2^H NMR),^22–24^ or for example Fourier-transform infrared spectrometry (FTIR),^25–27^ whereby, e.g., statements regarding the lamellar phase behavior or the lateral ordering of the SC lipids are possible. The latter is described to be liquid, hexagonal, or orthorhombic, depending on the distance between the lipid head groups, the lipid chain lengths, and their corresponding packing.^15,28,29^ At a skin temperature of 32 °C, the SC lipids are densely packed (orthorhombic), while both the hexagonal (medium packed) and even the disordered (liquid-like) phases appear in small ratios in SC lipid models.^30,31^ The extent of the orthorhombic phase was described in direct relation to the transepidermal water loss (TEWL) through the SC: due to the tight packing of the lipids in an orthorhombic phase, the water loss was limited.^32^ In turn, the liquid phase was described to be highly permeable.^33^ So, it is not surprising that the orthorhombic phase was found to be dominating in native and healthy human SC to ensure an intact barrier function.^29^

Thus, CERs were known to be dominating the arrangement of the SC lipid matrix due to immense nonpolar interactions of their carbon chains together with the free fatty acids.^8^ Furthermore, hydroxylation, i.e., the number of hydroxyl (OH) groups, also influences the arrangement of the CERs,^34–36^ as they form hydrogen bonds among themselves, which are preferentially found between the OH groups and the amide function of the headgroup. Serving as both hydrogen bond acceptors and donors,^37^ phytosphingosine CERs were described to form stronger hydrogen bond networks (HBNs) than sphingosine CERs.^34,38^ Moreover, not only the sphingoid base and its number of OH groups are important but also the kind of the *N*-acylated fatty acid. Thus, e.g., CER­[NS] was discussed to be responsible for the transversal bonding and with it for the integrity within each lipid layer (interlamellar), while its α-hydroxylated counterpart CER­[AS] was more supporting a cohesive lamellar structure due to an intralamellar orientation of the hydrogen bond network between different lipid layers.^39,40^ Based on the presence of HBNs, it is not surprising that the head groups of CERs can be hydrated. The water predominantly binds to the carbonyl oxygen function of the head groups via hydrogen bonds,^41^ although it has to be mentioned that only a minimal amount of water was described to be bound there.^42–44^ However, the bonds are not a rigid construct but fluctuate continuously. In a former work, Rerek et al. determined the effects of the head groups of CER­[NP], [AP], [NS], and [AS] by FTIR spectroscopy.^38^ In this study, they even found sphingosine-based CERs to be responsible for the orthorhombic chain packing and to be the driving force for the self-assembling process in the presence of other SC lipid components.^31,45^ A pioneer work of Löfgren and Pascher from 1977 also found CER­[NS] to form a close-packed arrangement with vertically directed chain orientation,^46^ when they investigated the CER monolayer and described the corresponding pressure/area isotherms. Besides, it is known that phytosphingosine CERs occupy a larger area per molecule than the sphingosine CERs, and the chain packing and the conformational chain order were also found to be different between these two CER subspecies.^47^ Compared to the orthorhombic packing of the sphingosine CER­[AS] and CER­[NS], the phytosphingosine CER­[AP] and CER­[NP] exhibit a less ordered, hexagonal packing of the CER chains.^48^ Although the sphingosine and phytosphingosine CERs have been studied very detailed over the last decades as mentioned earlier, both dihydrosphingosine CERs [AdS] and [NdS] were not fully characterized yet with respect to their single lipid phase behavior and interactions on the molecular level, besides Löfgren and Pascher, who described CER­[NdS] to contain a larger area per molecule and more disordered chains compared to its counterpart CER­[NS].^46^ The latest findings were made in SC model lipid mixtures presented by Školová et al., who described no distinct effect of the trans double bond in CER­[NS] compared to [NdS] and suggested that dihydrosphingosine CERs seemed to be underestimated SC components with respect to a distinct function.^49^ Moreover, their work from 2021 investigated the phase transition and permeability in SC lipid models, where especially for the latter, the isomeric forms of CER­[AdS] were described to act differently and had even a twice higher permeability compared to the CER­[NdS] membrane.^50^

But, as CERs are affected by the presence of cholesterol and free fatty acids in complex SC lipid model mixtures,^51^ it is highly important to focus on the single CER compounds. In the present work, we investigated the nature and extent of the influence of small structural differences in the head groups of the selected CERs, in detail double bonds and number and position of hydroxyl groups, on the lateral packing behavior of the pure CERs in monolayers using the Grazing Incidence X-ray Diffraction (GIXD) technique. In order to clearly identify the effect of the distinct headgroup, the CERs [AP], [NP], [AS], [NS], [AdS], and [NdS] with symmetric chain lengths of C18 were used as single lipids. In this way, we aim to gain a better insight into the molecular interactions and the phase behavior of the individual CERs depending only on their headgroup structure.

## Experimental Section

### Materials

All CER subclasses investigated have a symmetric chain length of C18 of both sphingoid base and fatty acyl. The CERs [AS], [AP], [NS], and [NP] were kindly provided by Evonik Industries AG, Essen, Germany. As for the D-conformation (with respect to the chiral center at the fatty acyl chain) of CER­[AP], a phase behavior was reported, which is comparable to that of native SC lipids;^21^ this was also assumed for CERs D-[AdS] and D-[AS]. Therefore, all α-hydroxylated CERs were used in their D-conformation for better comparability. The diastereoisomeric mixtures of the CERs [AS] and [AP] were stereoisomerically separated following a previously described procedure.^21^ CER­[NdS] and D-CER­[AdS] were obtained by a direct acylation of dihydrosphingosine with appropriate acid (stearic or racemic 2-hydroxystearic) in the presence of *N*-(3-(dimethylamino)­propyl)-*N*′-ethylcarbodiimide and 1-hydroxybenzotriazole hydrate followed by a chromatographic separation of diastereomers in the case of CER­[AdS]. Thereby, the spectral data of CER­[NdS] were in accordance with the published literature.^52^ The synthesis of CER­[AdS] was performed following ref 50 and the corresponding NMR characterization can be found in the Supporting Information. Chloroform was purchased from Carl Roth GmbH, Karlsruhe, Germany, and methanol was from VWR International, Radnor, USA.

### Methods

#### Pressure–Area Isotherms on the Aqueous Subphase

The CERs were dissolved in a chloroform/methanol mixture (3:1, *v*/*v*) with a final concentration of 1 mg/mL, and using a Hamilton syringe (100 μL syringe model 1810, Hamilton Bonaduz AG, Bonaduz, Switzerland), 20 μL of each CER solution was spread onto the air–water interface (160 cm^2^) of a Langmuir trough device (Riegler & Kirstein GmbH, Potsdam, Germany). The pressure–area isotherms were recorded at room temperature (20 °C). As pressure sensor, a Wilhelmy paper plate was used. After the evaporation of the organic solvent (about 10 min), the formed monolayers were laterally compressed to the target pressure with a compression speed of ∼4.4 Å^2^/(molecule·min). Three independent isotherm measurements (*n* = 3) were carried out and averaged for each individual CER component. Since not all CERs were compressible to 30 mN/m (a monolayer pressure of 30–35 mN/m is often assumed as an equivalent bilayer pressure^53,54^ ), the area values at 20 mN/m were considered for comparison. The area compression modulus *K*_a_ of a CER monolayer was determined from the slope of the isotherm and the available molecular area *A*_mol_^a^ at 25 mN/m using eq 1.

1 Ka=−Amola(dIIdAmola)

#### Grazing Incidence X-ray Diffraction

GIXD experiments were performed at the beamline P08 at the PETRA III synchrotron (Deutsches Elektronen-Synchrotron, DESY, Hamburg, Germany)^55^ using the standard configuration of the Langmuir grazing incidence diffraction setup.^56^ While the details of the setup have been described elsewhere,^56^ key parameters are listed here. The incident beam energy was 15 keV, corresponding to a wavelength of 0.827 Å, and the beam size was 70 μm (vertical) × 1000 μm (horizontal). The incident angle of 0.07° corresponds to 85% of the critical angle of the air–water interface and provides a footprint of 1 × 50 mm^2^. A glass plate was placed approximately 0.3 mm beneath the illuminated area of the monolayer in order to suppress the mechanically excited vibrations of the water surface. The Langmuir trough (Kibron Inc., Helsinki, Finland) was kept at 20 °C by a thermostat and enclosed in a prehumidified helium atmosphere to reduce the air scattering background and the radiation-induced damage. The diffraction signal was measured by a MYTHEN2 1K detector (DECTRIS, Baden, Switzerland) placed after a Soller collimator (JJ X-Ray, Denmark) to provide an in-plane angular resolution of 0.09° (full-width-at-half-maximum, fwhm).^57–59^ Model peaks taken as Lorentzian in the in-plane (Bragg peaks, *Q*_*xy*_) and as Gaussian in the out-of-plane direction (Bragg rods, *Q*_*z*_) were fitted to the integrated intensities. The obtained Bragg peak and Bragg rod positions give information about the monolayer structure, such as the tilt of the chains (*t*), lattice distortion (*d*), cross-sectional area (*A*_0_), and the in-plane lattice area per chain (*A*_*xy*_). The thickness of the monolayer part contributing to the scattering signal was estimated from the full-width at half-maximum (fwhm) of the Bragg rod using the Scherrer equation (eq 2).^60,61^

2 $$L_{z}∼0.9\frac{2\pi }{fwhm\;\left(Q_{z}\right)}$$

## Results and Discussion

### Pressure–Area Isotherms of Single CERs on the Aqueous Subphase

Experiments were performed at 20 °C to minimize water evaporation, which is especially important for GIXD experiments. As shown earlier,^51,62^ the increase to normal skin temperature (32–34 °C)^63^ neither changes the CER pressure/area isotherms nor the phase state both in pure CER monolayers and/or in bulk systems. The transition/melting temperatures of ceramides are known to be very high. For all isotherms, only the transition from a gas-analogue state to a condensed phase with dense packing (resublimation) can be observed at very low or vanishing lateral pressure (see Figure 2). This result confirms earlier reports of condensed CER monolayers, but mainly for lipid mixtures.^64,65^ Based on the GIXD data described further below, the areas per molecule determined from experimental isotherms deviate in average by 10% from the molecular areas determined from GIXD. The same applies for the comparison with literature data.^46^ Therefore, let us discuss some of their drawbacks that make it difficult to obtain reliable and precise isotherms besides the usual challenges like exact concentration determination and spreading. One additional problem with CERs is their solubility. In many cases, it is difficult to find an appropriate solvent that can be used safely as a spreading solvent for monolayer preparation. Therefore, the concentration in the spreading solution could be smaller than calculated, which would shift the isotherm to smaller apparent areas. This is not observed for the CERs used (see Figure 2), and GIXD experiments show no evidence of bulk material at the air/water interface. The apparent molecular areas of CER­[NdS], CER­[AdS], CER­[NP], and CER­[AP] are slightly too large in light of tight packing of chains in the all-trans conformation with a cross-sectional area of around 20 Å^2^. But this could be also a result of tilting the chains. The head groups are small and therefore should have no major influence on the packing density. Only the molecular areas of CER­[NS] and CER­[AS] are smaller than expected. Additionally, the apparent slopes of the isotherms of CER­[NdS] and CER­[AdS] are clearly too small for condensed isotherms. The reason for this problem could be the stiffness of the monolayers because CERs can form head-to-head hydrogen bonds^37^ that promote strong intermolecular interactions in biological membranes. Therefore, CERs in lipid membranes frequently segregate into ordered domains with gel- or solid-like character.^66^ If the paper Wilhelmy plate method is used, a meaningful pressure recording of such layers is sometimes difficult, as CER films are not easily compressible.^67^ The stiffness of the plate leads to perturbed pressure measurements with poor reproducibility concerning especially the lateral compression modulus *K*_a_, extracted from the experimentally observed isotherms, as already described for similar systems.^68^ Löfgren and Pascher^46^ used a newly constructed surface balance based on the technique of Langmuir and Adams. The Wilhelmy method with the vertically suspended plate proved less reliable, as rigid monolayer states give a poorly defined contact angle between the plate and the water surface. Comparing this data with our, obtained with the Wilhelmy plate, indicates that especially the slope of the isotherms is different.

**Figure 2:**
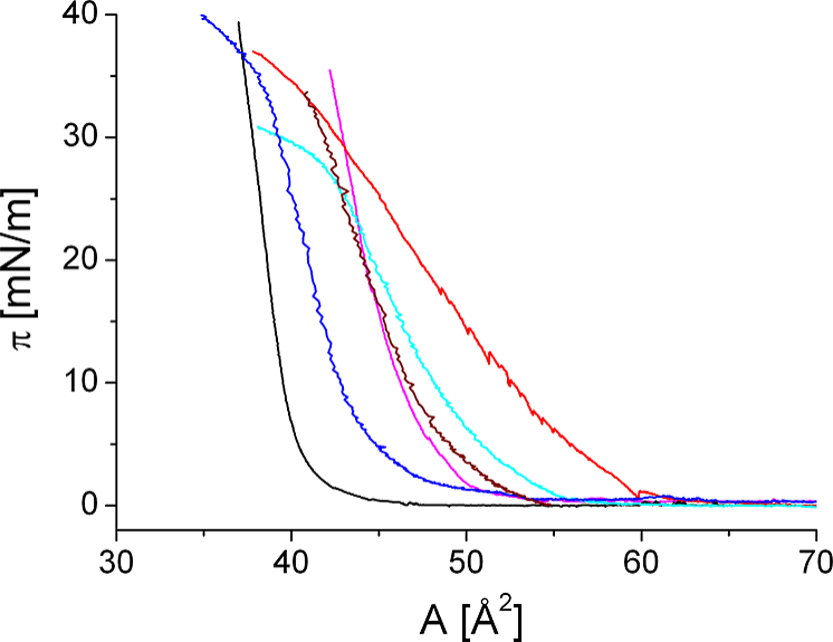
Isotherms of the studied single short-chain CERs: CER­[NdS]—red, CER­[AdS]—cyan, CER­[NP]—brown, CER­[AP]—magenta, CER­[NS]—black, and CER­[AS]—blue.

From the slopes of the pressure–area isotherms, the area compression modulus *K*_a_, which is the reciprocal of the compressibility and equivalent to elasticity, is obtained according to eq 1 (see Table 1). Typical values for *K*_a_ of DPPC (1,2-dipalmitoyl-phosphatidylcholine, a standard phospholipid in membranes) monolayers are in the range of 10–50 mN/m in the LE (liquid-expanded) phase and 100–250 mN/m in the liquid-condensed (LC) phase.^69,70^ In the hexagonal phase (LS) of the single-chained octadecanol monolayer, even *K*_a_ values above 1000 mN/m have been observed.^71^ The compression modulus is practically a spring constant, representing the stiffness of the monolayer. The larger the value of *K*_a_, the stiffer the layer, and a larger force is needed to compress it. This is especially expected for highly condensed monolayers forming an intermolecular HBN such as CER­[NP] and CER­[AP] (see GIXD data in Figures 4C and 5C).

**Table 1:** Area per Molecule Extracted from the Isotherms at 20 and 30 mN/m as Well as the Area Compression Modulus *K*_a_ of Studied CER Monolayers^a^

				values from the literature^46^	
compound	area at 20 mN/m [Å^2^]	area at 30 mN/m [Å^2^]	*K*_a_ [mN/m]	area at 30 mN/m [Å^2^]	*K*_a_ [mN/m]
CER[NdS]	47.3 ± 0.7	42.7* ± 0.4	98 ± 8	48.0	172
CER[AdS]	44.6 ± 0.6	41.4* ± 0.6	135 ± 8	45.0	213
CER[NP]	44.1 ± 0.6	41.0 ± 0.7	200 ± 10	44.0	345
CER[AP]	44.2 ± 1.0	42.9 ± 0.7	335 ± 10	44.0	303
CER[NS]	38.5 ± 0.4	37.8 ± 0.4	545 ± 10	40.5	1000
CER[AS]	40.9 ± 1.6	39.2 ± 1.2	235 ± 10	41.0	625

a *Indicates extrapolated values (*n* = 3).

With 545 mN/m, CER­[NS] had the highest area compression modulus and CER­[NdS] the smallest one with 98 mN/m, whereby for CER­[AdS], a similar but too small value (similar to LE phase) was found. This observation might be due to the stiffness of these monolayers, leading to the tilting of the Wilhelmy plate and therefore to apparently smaller slopes of the isotherms. Therefore, the isotherm of CER­[NdS] has also been measured during expansion (see Figure 3).

**Figure 3:**
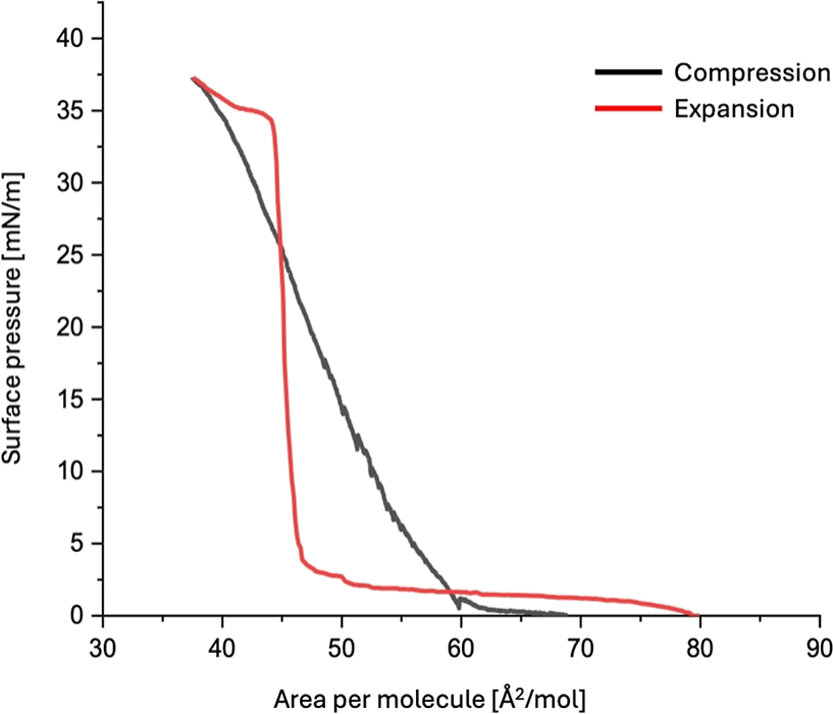
Isotherms of CER­[NdS] measured during compression (black) and expansion (red).

The huge difference between compression and expansion is typical for tilting of the Wilhelmy plate. During expansion, the plate returns to the vertical position, and only then can correct pressure values be determined. Then, a typical value for LC phases (560 mN/m) of the compression modulus *K*_a_ has been observed. In average, our *K*_a_ values are always smaller than the ones obtained by Löfgren and Pascher^46^ but at most by a factor of 2.

A possible reason for observing too large molecular areas of CERs [NdS], [AdS], [NP], and [AP] may be that monolayers undergo the direct transition from the gaseous phase to a condensed phase (resublimation), whereby the condensed islands are already formed during spreading. Before the lift-off point, the solid phase coexists with the gaseous phase between the condensed islands. Defects in the condensed monolayer will depend on the solvent used and the spreading conditions, but such defects are usually in the range below 5% of the total condensed area but have a huge influence on the compression modulus *K*_a_ in comparison with modulus *K*_c_ determined from X-ray data.^71^ During compression, the percentage of the gaseous phase decreases gradually. However, it might be possible that some parts of the gaseous phase remain trapped between the highly condensed islands. A similar unusual behavior has been described for a triple-chain phospholipid 1­(2C_16_-18:0)-2H-PE, showing large differences between the molecular areas derived from isotherm and X-ray measurements.^72^ The tilting of the triple-chain molecules led to an orientational ordering of the headgroup dipoles and therefore to an electrostatic repulsion between condensed phase domains. This repulsion between condensed phase domains has been discussed to be the reason for the formation of holes in the monolayer. In the present case of CERs, the extremely tight packing in the condensed phase domains might be the reason for the formation of holes. The trapped gaseous phase exists along the isotherm, and a very long time is needed to anneal such a layer.

### Grazing Incidence X-ray Diffraction

The X-ray intensities as a function of the in-plane (*Q*_*xy*_) and out-of-plane (*Q*_*z*_) components of the scattering vector are presented in Figure 4 for the studied nonhydroxy CERs. For CER­[NS] (Figure 4A), just above the lift-off point (the molecular area at the surface pressure rise), two diffraction peaks with out-of-plane maxima at *Q*_*z*_(1) = 0 and *Q*_*z*_(2) > 0 (0.33 Å^–1^) were detected, characteristic of an orthorhombic unit cell with NN (nearest neighbor) tilted alkyl chains. In the in-plane direction, these peaks were located at *Q*_*xy*_(1) = 1.517 Å^–1^ and *Q*_*xy*_(2) = 1.469 Å^–1^. The corresponding lattice parameters (deduced from the peak positions as described in the Methods section) are presented in Table S2 in the Supporting Information. The tilt angle of the chains amounted to 14.7°. The cross-sectional area of one chain was *A*_0_ = 20.0 Å^2^, typical for phases with free rotation of the chains. Such an orthorhombic unit cell was also observed in native SC material and has been discussed to be mandatory for an intact SC barrier function.^32^

**Figure 4:**
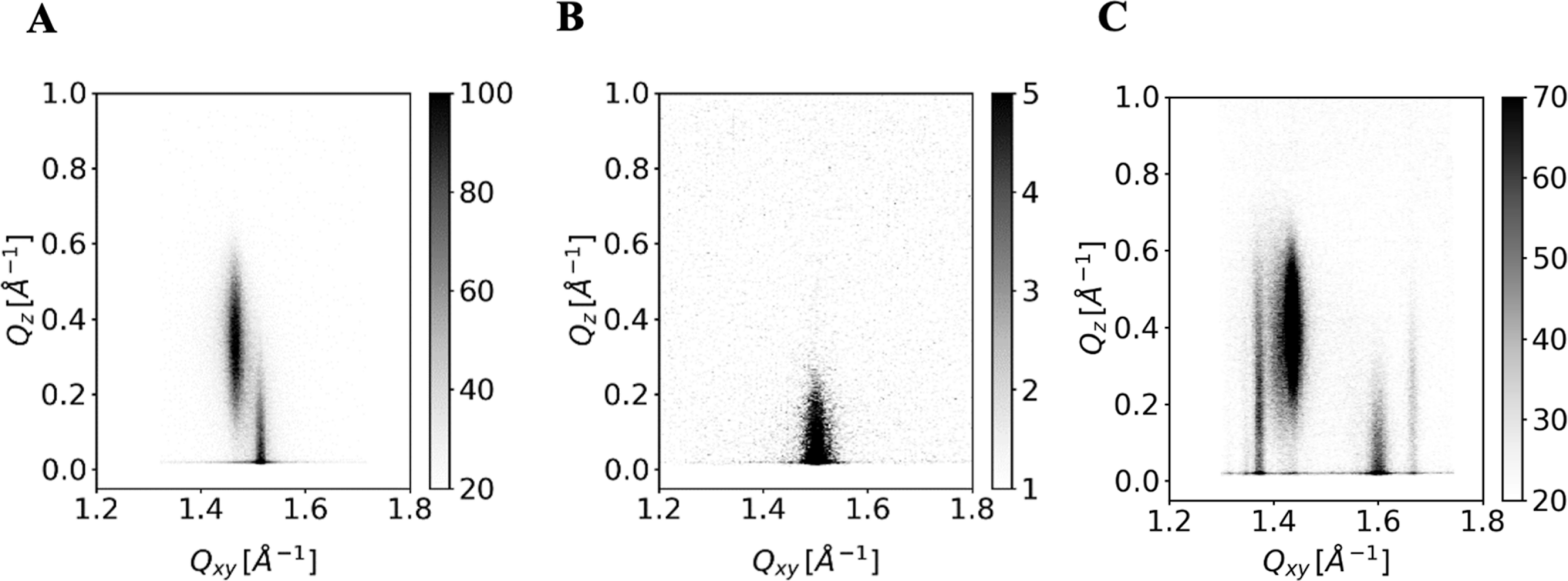
GIXD pattern (intensity vs *Q*_*xy*_ and *Q*_*z*_) of a CER­[NS] (A), CER­[NdS] (B), and CER­[NP] monolayer (C) at π = 10 mN/m.

The very small structural difference between CER­[NS] and CER­[NdS] is the lacking double bond at C4 of the latter, which has immense impact on the GIXD intensity pattern *I* (*Q*_*xy*_, *Q*_*z*_) (see Figure 4B): the only Bragg peak seen was located at *Q*_*xy*_ = 1.505 Å^–1^ and *Q*_*z*_ = 0 Å^–1^. The chains are nontilted and packed in a hexagonal lattice. With 20.1 Å^2^, the cross-sectional area was marginally larger compared with CER­[NS] showing that the double bond at C4, close to the headgroup, hardly influences the packing density in the monolayer.

The headgroup of CER­[NP] contains one additional functional hydroxyl group (three instead of two OH groups compared to CER­[NS] and CER­[NdS]). So, it was not surprising that the diffraction pattern of CER­[NP] was found to be much more complex and exhibited several Bragg peaks (Figure 4C). The regions in which the Bragg peaks, originating from the chain packing, were expected were analyzed, and the results are presented in Figure S1 and Table S1 in the Supporting Information. The three main peaks at *Q*_*xy*_ positions of 1.373, 1.439, and 1.667 Å^–1^ belong to the chain lattice and describe an oblique unit cell with tilted chains (*t* = 15.6°). Based on the determined chain lattice parameters (see Table S2 in the Supporting Information), a commensurate unit cell of a supramolecular lattice (*a*_s_ = 9.148 Å, *b*_s_ = 9.588 Å, *y* = 107.3°) could be constructed. This lattice is an integer multiple of the basis vectors of the smaller sublattice.

The unit cell with an area of 83.7 Å^2^ accommodates two CER­[NP] molecules. The corresponding Bragg peak positions have been calculated and compared with the experimentally found ones, proving the correctness of the assumed supramolecular lattice (see Table S2). The only exceptions were a broad and weak peak at 1.544 Å^–1^ and a quite pronounced Bragg peak at 1.6 Å^–1^, which did not belong to the CER­[NP] supramolecular lattice.

The pressure-independent peak positions indicate that the unit cell dimensions are very rigid and incompressible, which in turn indicates that it is stabilized by the molecular superlattice that might be originated from the intermolecular HBN between the head groups.^73^ Thus, it has to be underlined that OH groups are predestined for intermolecular hydrogen bonding, and such HBN will surely rigidify the monolayer, which was described earlier as well.^35^ With *A*_0_ = 20.2 Å^2^, the cross-sectional chain area is again typical for rotator phases (free rotation of the chains) in phospholipid monolayers.^59^ In the case of CER­[NP], we were able to perform GIXD experiments at two different lateral pressures. This allows us to calculate the diffraction-based lateral compression modulus *K*_c_ using the pressure dependence of the Bragg peak position (*Q*_*xy*_^max^) with eq 3.^71^

3 $$K_{c}=\frac{Q_{xy}^{\max}}{2}\times \frac{d\pi }{dQ_{xy}^{\max}}$$

In hexagonal phases, this can be directly determined via the change in the position of the diffraction peak during compression. In phases with a rectangular or oblique unit cell, the lateral compression modulus *K*_c_ along each diffraction vector *Q*_*hk*_ can be determined as *K*_c_(*hk*) with eq 4.^74,75^

4 $$K_{c}\left(hk\right)=\frac{{[Q}_{xy}^{\max}]\left(hk\right)}{2}\times \frac{d\pi }{d{[Q}_{xy}^{\max}]\left(hk\right)}$$

For monolayers undergoing the direct transition from the gaseous phase to a condensed phase (resublimation), the condensed islands are already formed during spreading. The compression moduli based on diffraction experiments *K*_c_(π) and based on isotherm measurements *K*_a_(π) are usually extremely different (factor of 25), and *K*_c_ does practically not change with compression, indicating that the packing density of such condensed layers is almost not influenced by compression as already described for octadecanol monolayers in the isotropic LS phase.^74^ Obviously, the defects produced during the spreading procedure have a large influence on the isotherm-based compression modulus *K*_a_. The three *K*_c_ values determined for CER [NP] based on GIXD experiments are 2750, 960, and 3335 mN/m. The corresponding *K*_a_ values are 200 mN/m (our data) and 345 mN/m.^46^ Let us take the more trustful values of Löfgren and Pascher;^46^ the *K*_c_ values are only 3–10 times larger than *K*_a_. With the data presented here, the factors *K*_c_/*K*_a_ are between 5 and 17.

The studied nonhydroxy CERs were also used earlier, where a decreased tendency of orthorhombic packing from CER [NS] over CER [NdS] to CER [NP] in more complex SC lipid mixtures was found.^35,76^ A reason for the rigidity, especially of the CER­[NS] monolayer observed in the study presented, could be a very high crystalline and rigid ratio of the CER, as Stahlberg et al. revealed by deuterium solid-state NMR spectroscopy for a SC lipid mixture.^77^ Compared to that, they found CER­[NP] to form predominantly gel and fluid phases, where they discussed packing defects due to the OH group at C4 of the phytosphingosine moiety.

The contour plots of the X-ray intensities as a function of the in-plane (*Q*_*xy*_) and out-of-plane (*Q*_*z*_) components of the scattering vector of the α-hydroxy CERs studied are presented in Figure 5.

**Figure 5:**
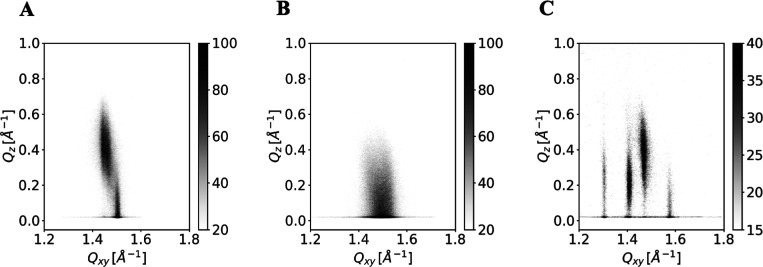
GIXD pattern (intensity vs *Q*_*xy*_ and *Q*_*z*_) of a CER­[AS] (A), CER­[AdS] (B), and D-CER­[AP] (C) monolayer at π = 10 mN/m.

For CER­[AS], the additional OH group in the α-position at the fatty acyl chain leads to stronger distortion of the monolayer lattice. Just above the lift-off point, three Bragg peaks were clearly identified (Figure 5A). The Bragg peak (*Q*_*xy*_) and Bragg rod (*Q*_*z*_) positions are listed in Table S2. These three peaks correspond to an oblique structure of the condensed monolayer. With 18.2°, the tilt angle is markedly larger compared with that of CER­[NS]. Obviously, the OH group in the α-position influences the packing in the monolayer. To maximize the van der Waals interactions between the chains and keeping the preferred packing density (*A*_0_ = 20.0 Å^2^), the chains have to tilt more strongly and therefore distort the lattice from orthorhombic to oblique.

Compared to CER­[AS], the lack of a double bond in CER­[AdS] leads to a scattering pattern with two Bragg peaks close to the horizon (Figure 5B). In contrast to CER­[NdS], which forms a hexagonal lattice of nontilted chains (one Bragg peak), only two Bragg peaks describe the experimentally observed GIXD intensity distribution satisfyingly. The two Bragg peaks are very close to each other (*Q*_*xy*_(1) = 1.516 Å^–1^, *Q*_*z*_ (1) = 0 Å^–1^ and *Q*_*xy*_(2) = 1.469 Å^–1^, and *Q*_*z*_(1) = 0.28 Å^–1^). The cross-sectional area (*A*_0_ = 20.2 Å^2^) was again only marginally larger than for CER­[AS] (*A*_0_ = 20.0 Å^2^), demonstrating that the double bond at C4 close to the headgroup does almost not influence the packing density in the monolayer as already observed for the nonhydroxy CERs. With ∼12°, the tilt angle of the chains is quite small.

The 2D structures of the two separated diastereomers of CER­[AP] with symmetric (C18:C18) alkyl chains were studied previously and described in ref 78. The contour plot of the D-CER­[AP] was characterized by several Bragg peaks, as found and described in this work for CER­[NP]. The three main peaks (Figure 5C) are characteristic for an oblique unit cell with tilted chains (*t* = 14.0°). Based on the determined chain lattice parameters, a larger unit cell of a supramolecular lattice (*a*_s_ = 9.334 Å, *b*_s_ = 9.746 Å, γ = 113.7°) originating from intermolecular HBN between the head groups has been assumed.^78^ The unit cell with an area of 83.3 Å^2^ accommodates 2 D-CER­[AP] molecules. The corresponding Bragg peak positions have been calculated and compared with the experimentally found ones (see Table S3 in the Supporting Information). Increasing the surface pressure again did not affect the unit cell dimensions. The cross-sectional chain area *A*_0_ was the same as that found for CER­[NP] (*A*_0_ = 20.2 Å^2^). Obviously, not only the number but also the steric orientation of the OH groups is important for the formation of an intermolecular HBN between the head groups, which was observed for both phytosphingosine CERs [NP] and [AP].

In contrast, L-CER­[AP] displayed only three Bragg peaks describing an oblique unit cell of tilted chains (*t* = 20.2°), and no additional peaks representative of a molecular superlattice due to HBNs have been observed.^78^ The fwhm of the Bragg rods (peaks along the *Q*_*z*_ direction) allowed estimation of the length of the scattering unit (the stretched alkyl chains). The theoretical length of a chain (*L*) in the all-trans conformation can be calculated by *L* = n*1.25 + 1.54 [Å], with *n* = number of methylene groups.^79^ A C18 chain in the all-trans conformation should have a length of ∼23 Å. The effective thickness of the chain region estimated from the fwhm of the Bragg rods lies between 19 Å for CER­[AS], CER­[AdS], CER­[NS], and CER­[NP], 21 Å for CER­[AP], and 23 Å for CER­[NdS], demonstrating that almost the whole chains are completely stretched and contribute to the diffraction peaks, especially in the case of CER­[NdS].

## Conclusions

Our GIXD experiments have clearly demonstrated that the phase behavior of CERs is strongly influenced by the chemical structure of the headgroup, even if these differences are quite small and merely concern the number and positions of OH groups or a double bond close to the headgroup. One important finding is that the HBNs in the CER­[NP] and D-CER­[AP] monolayers were formed only in the presence of the OH group at C4 of the phytosphingosine moiety. The HBN determines the packing and distortion of the CER chains. Such HBNs remain stable against increasing lateral pressure and prevent structural changes of the monolayers confined to the planar air/liquid interface. However, the packing density of the chains is only insignificantly smaller than that in the corresponding monolayers of CERs with sphingosine moieties. The present results are in line with the work of Rerek et al.,^38^ who found in supported hydrated films that the relative contributions of chain packing and H-bonding under physiological conditions differ markedly for phytosphingosine CERs compared to sphingosine CERs. The phytosphingosine CERs are characterized by hexagonal chain packing with relatively strong H-bonding and the sphingosine CERs by orthorhombic chain packing and weaker H-bonding. Thus, together with their relative abundance, which is in sum 30–40% of all CERs in human SC,^9,13,80^ we have distinct hints that CER­[NP] and CER­[AP] are powerful components underlining their essentiality for an intact skin barrier, whereas CER­[AP] seems to form even more rigid layers with respect to its *K*_a_. The demonstration that only the phytosphingosine CERs [AP] and [NP] can form supramolecular structures underlines the high importance of phytosphingosine CERs for an intact skin barrier function. In fact, it is known that as in diseased skin, the majority composition shifts from phytosphingosine CERs to sphingosine CERs together with decreased barrier properties.^1,4^

The absence of the OH-group and presence of the trans double bond at C4 as in sphingosine CERs influence the chain packing of the CERs due to less HBN forces (see also refs 38 and 46 ). This was recently described for complex SC lipid model mixtures as well, where changes in the headgroup structure of CERs influenced the lateral packing and the formation of HBNs.^26^ Here, GIXD revealed that the alkyl chains of both sphingosine CERs were freely rotatable, but the α-hydroxy group led to a larger lattice distortion (from orthorhombic packing observed for CER­[NS] to oblique) and to a higher tilt of the CER­[AS] chains.

For the dihydrosphingosine CERs [AdS] and [NdS], the effect of the α-hydroxy group on the chain tilt is much stronger due to the missing double bond. CER­[NdS] is the only compound in this study that is able to form a layer with hexagonal packing of nontilted chains. Due to the stiffness of the layers leading to an undefined tilt of the Wilhelmy plate, trustful isotherms are missing. Therefore, measuring decompression isotherms is very important. Such experiments performed with CER­[NdS] show convincingly that these films are highly rigid with large compression modules what could be expected based on GIXD data. Altogether, the presented data reveal an important role of dihydrosphingosine CERs in the barrier function of an intact SC.

## Supplementary Material



## Notes

Experimentally observed GIXD profile (scattering intensity vs *Q*_*xy*_) of the CER­[NP] monolayer, positions with Miller indices of the calculated Bragg peaks together with the experimentally observed scattering profile of CER­[NP] monolayers at 20 °C and 10 mN/m, Bragg peak positions (*Q*_*xy*_, Å^–1^) and the corresponding full-widths at half-maximum (fwhm) determined from the GIXD data of the CER­[NP] monolayer, Bragg peak (*Q*_*xy*_) and Bragg rod (*Q*_*z*_) positions and the corresponding fwhm of the CER monolayers as well as their corresponding lattice parameters at different lateral pressures and 20 °C, and in-plane peak positions and calculated Miller indices for CER­[NP] and D-CER­[AP] NMR characterization of the CER­[AdS] (PDF)

## #

Pharmacy, Leipzig University Medical Center and Medical Faculty, Liebigstr. 20, 04103 Leipzig, Germany

## Abbreviations

AdS: α-hydroxy-dihydrosphingosine
AP: α-hydroxy-phytosphingosine
AS: α-hydroxy-sphingosine
CER: ceramide
DESY: Deutsches Elektronen-Synchrotron
DPPC: 1,2-dipalmitoyl-phosphatidylcholine
FTIR: Fourier-transform infrared spectroscopy
fwmh: full-width-at-half-maximum
GIXD: grazing incidence X-ray diffraction
HBN: hydrogen bond network
^2^H NMR: deuterium nuclear magnetic resonance
*K*_a_: isotherm-based lateral compression modulus
*K*_c_: diffraction-based lateral compression modulus
NdS: nonhydroxy dihydrosphingosine
NP: nonhydroxy phytosphingosine
NS: nonhydroxy sphingosine
NN: nearest neighbor
OH group: hydroxyl group
*Q*_*hk*_: diffraction vector
SC: Stratum corneum
