# Supplementary material for: Structure–Function Relationship of the Most Abundant Ceramide Subspecies Studied on Monolayer Models Using GIXD and Langmuir Isotherms
Source: Langmuir. 2025 May 27;41(22):14255–64. doi: 10.1021/acs.langmuir.5c01340 (PMC12164350; doi:10.1021/acs.langmuir.5c01340)
Supplement: Supplementary file 1 [file la5c01340_si_001.pdf]

Supporting Information

Structure-function relationship of the most abundant ceramide subspecies studied on monolayer models using GIXD and Langmuir isotherms

Gerald Brezesinski [1,2,‡], Lukáš Opálka [3‡], Chen Shen [4], Carolin Groetzsch [1,†], Emanuel Schneck [2], Adina Eichner [1,5]*

[1] Institute of Applied Dermatopharmacy at Martin Luther University Halle-Wittenberg, Weinbergweg 23, 06120 Halle (Saale), Germany

[2] Institute for Condensed Matter Physics, Technical University of Darmstadt, Hochschulstr. 8, 64289 Darmstadt, Germany

[3] Skin Barrier Research Group, Faculty of Pharmacy, Charles University, Heyrovského 1203, 500 05 Hradec Králové, Czech Republic

[4] Deutsches Elektronen-Synchrotron DESY, Notkestr. 85, 22607 Hamburg, Germany

[5] Department of Dermatology and Venereology, Martin Luther University Halle-Wittenberg, Ernst-Grube-Str. 40, 06120 Halle (Saale), Germany

GIXD analysis of CER[NP]

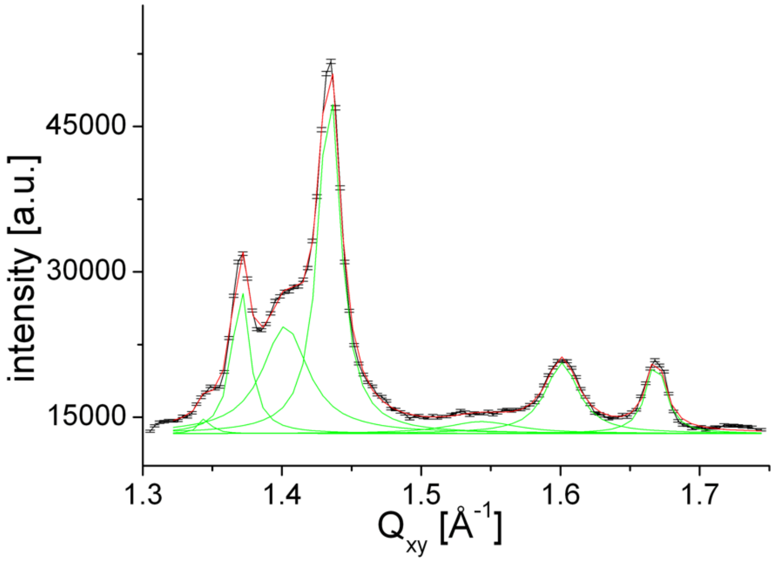

Figure S1. Experimentally observed GIXD profile (scattering intensity vs. Q_{xy}) of the CER[NP] monolayer with symmetric C18 chains at 10 mN/m and 20 °C with the corresponding fit (green).

Table S1. Bragg peak positions (Q_{xy}, Å^{-1}) and the corresponding full-widths at half-maximum (*fwhm)* determined from the GIXD data of the CER[NP] monolayer with symmetric C18 chains presented in Fig. S1.

Q_{xy}<br>fwhm	1.345<br>0.010	1.373<br>0.018	1.402<br>0.040	1.439<br>0.024	1.544<br>0.061	1.601<br>0.029	1.667<br>0.018

GIXD data and corresponding structural parameters

Table S2. Bragg peak (Q_{xy}) and Bragg rod (Q_z) positions and the corresponding fwhm of the CER monolayers with symmetric C18 chains as well as the corresponding lattice parameters of the monolayers at different lateral pressures and 20 °C

π<br>[mN/m]	$Q_{xy}(1)$<br>[Å^{-1}]	$Q_z(1)$<br>[Å^{-1}]	$Q_{xy}(2)$<br>[Å^{-1}]	$Q_z(2)$<br>[Å^{-1}]	$Q_{xy}(3)$<br>[Å^{-1}]	$Q_z(3)$<br>[Å^{-1}]
**CER[NS]**						
5			1.469<br>0.037	0.33<br>0.29	1.517<br>0.022	0<br>0.29
**CER[NdS]**						
5					1.505<br>0.029	0<br>0.25
**CER[NP]**						

2	1.371<br>0.029	0.20<br>0.30	1.433<br>0.023	0.39<br>0.30	1.669<br>0.017	0.19<br>0.30
10	1.373<br>0.018	0.18<br>0.30	1.439<br>0.024	0.39<br>0.30	1.667<br>0.018	0.21<br>0.30
**CER[AS]**						
5	1.448<br>0.035	0.44<br>0.29	1.469<br>0.039	0.37<br>0.29	1.504<br>0.020	0.07<br>0.29
**CER[AdS]**						
10			1.469<br>0.084	0.28<br>0.30	1.516<br>0.061	0<br>0.30
**CER[AP]**						
20	1.408<br>0.024	0.20<br>0.27	1.470<br>0.026	0.36<br>0.27	1.575<br>0.022	0.16<br>0.27

π<br>[mN/m]	a, b, c<br>[Å]	α, β, γ<br>[°]	d	t<br>[°]	A_{xy}<br>[Å^2]	A_0<br>[Å^2]
**CER[NS]**						
5	4.994<br>4.836<br>4.836	117.8<br>121.1<br>121.1	0.043320	14.7	20.7	20.0
**CER[NdS]**						
5	4.821<br>4.821<br>4.821	120.0<br>120.0<br>120.0	0	0	20.1	20.1
**CER[NP]**						
2	4.584<br>4.792<br>5.581	128.2<br>124.8<br>107.0	0.247882	15.7	21.0	20.2
10	4.574<br>4.794<br>5.553	128.2<br>124.6<br>107.3	0.242602	15.6	20.9	20.2
**CER[AS]**						
5	4.841<br>4.911<br>5.028	121.7<br>120.3<br>117.9	0.044426	18.2	21.0	20.0
**CER[AdS]**						
10	4.993<br>4.838<br>4.838	117.9<br>121.1<br>121.1	0.042423	12.5	20.7	20.2
**CER[AP]**						
20	4.667<br>4.873<br>5.221	125.0<br>121.3<br>113.7	0.131995	14.0	20.8	20.2

Supramolecular monolayer structures of CER[NP] and CER[AP]

CER[NP] with symmetric chains forms an oblique chain lattice in monolayers. The presence of extra peaks indicates the formation of a supramolecular lattice (subgel phase) due to intermolecular hydrogen bond network (HBN). Lorentzian functions have been fitted to the experimentally observed scattering peaks. A supramolecular lattice with a_s = 9.148 Å, b_s = 9.588 Å, γ = 107.3° has been used to calculate the expected Bragg peak positions. The supramolecular unit cell accommodates 2 CER[NP] molecules.

In figure S2, the Miller indices of each Bragg peak are indicated. The calculated values are presented together with those of CER[AP] in table S3.

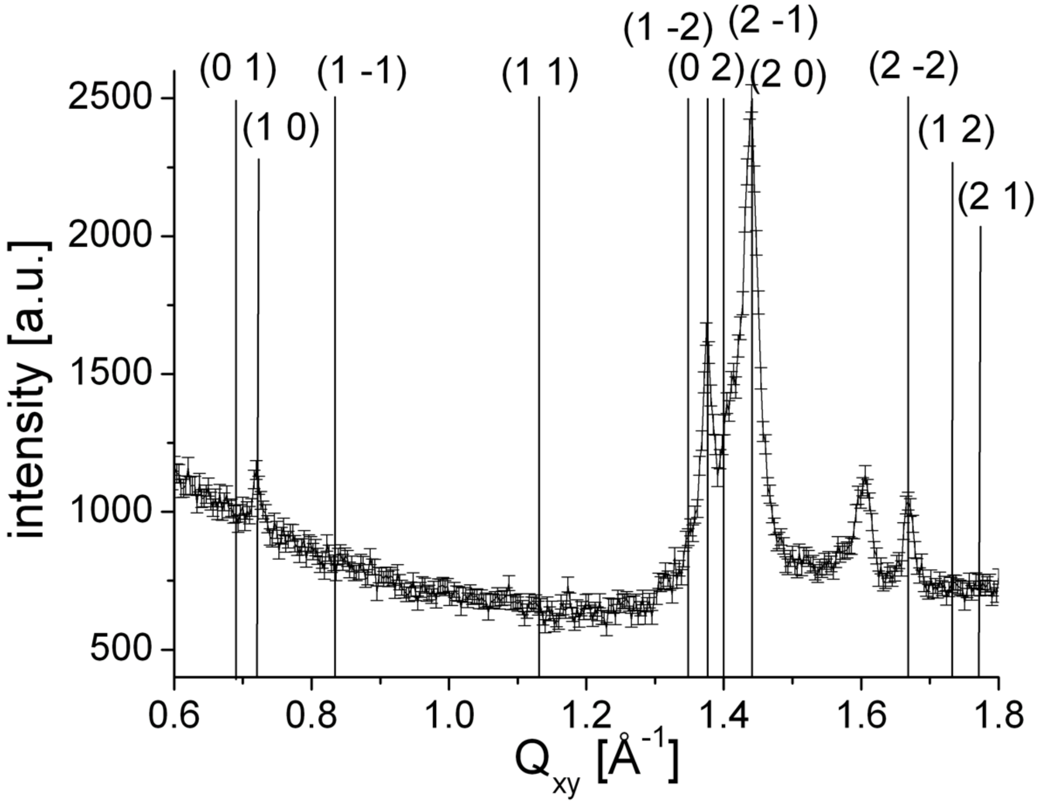

Figure S2. Positions with Miller indices of the calculated Bragg peaks (Table S3) together with the experimentally observed scattering profile of CER[NP] monolayers with symmetric chains 20 °C and 10 mN/m.

Table S3. In-plane peak positions and calculated (quadratic Bragg equation of a triclinic system) Miller indices for CER[NP] and D-CER[AP] in monolayers. A supramolecular lattice with a_s = 9.148 Å, b_s = 9.588 Å, γ = 107.3° has been used for CER[NP], and with a_s = 9.334 Å, b_s = 9.746 Å, γ = 113.7° for CER[AP]. The unit cell with an area of 83.7 Å^2 (CER[NP] or 83.4 Å^2 (CER[AP]) accommodates in both cases 2 CER molecules.

**CER[NP]**			**D-CER[AP]**		
Q_{xy} (measured) Å^{-1}	Q_{xy} (calculated) Å^{-1}	Miller Indices	Q_{xy} (measured) Å^{-1}	Q_{xy} (calculated) Å^{-1}	Miller Indices
	0.69	(0 1) + (0 -1)		0.70	(0 1) + (0 -1)
0.72	0.72	(1 0) + (-1 0)	0.74	0.74	(1 0) + (-1 0)
	0.83	(1 -1) + (-1 1)	0.79	0.79	(1 -1) + (-1 1)
	1.13	(1 1) + (-1 -1)		1.21	(1 1) + (-1 -1)
	1.35	(1 -2) + (-1 2)	1.30	1.30	(1 -2) + (-1 2)
1.37	1.37	(0 2) + (0 -2)		1.35	(2 -1) + (-2 1)
1.41	1.40	(2 -1) + (-2 1)	1.41	1.41	(0 2) + (0 -2)
1.44	1.44	(2 0) + (-2 0)	1.47	1.47	(2 0) + (-2 0)
1.67	1.67	(2 -2) + (-2 2)	1.58	1.58	(2 -2) + (-2 2)
	1.73	(1 2) + (-1 -2)	1.83	1.83	(1 2) + (-1 -2)
	1.77	(2 1) + (-2 -1)	1.87	1.87	(2 1) + (-2 -1)

NMR characterization of the CER[AdS]

^{1}H NMR (600 MHz, $CDCl_3$/MeOD 5:1) δ 3.97 (dd, J = 8.1, 3.7 Hz, 1H), 3.78 – 3.69 (m, 2H), 3.62 (dd, J = 11.2, 3.3 Hz, 1H), 3.56 – 3.50 (m, 1H), 1.77 – 1.46 (m, 2H), 1.47 – 1.29 (m, 4H), 1.18 (s, 52H), 0.80 (t, J = 7.0 Hz, 6H). ^{13}C NMR (151 MHz, $CDCl_3$/MeOD 5:1) δ 175.73, 72.51, 72.03, 61.44, 54.01, 34.39, 34.09, 31.91, 29.69, 29.65, 29.60, 29.50, 29.35, 25.86, 25.14, 22.66, 14.00.
